# A Population Survey on Barriers and Facilitators to Breast Cancer Screening Based on the Theoretical Domains Framework

**DOI:** 10.3390/bs15020209

**Published:** 2025-02-14

**Authors:** Sarah Huf, Ada Humphrey, Ara Darzi, Deborah Cunningham, Dominic King, Gaby Judah

**Affiliations:** 1 Department of Surgery and Cancer, Imperial College London, London SW7 2AZ, UKg.judah@imperial.ac.uk (G.J.); 2 Breast Surgery Unit, The Royal Marsden NHS Foundation Trust, Downs Rd, Sutton SM2 5PT, UK

**Keywords:** behavioural science, breast cancer, screening, public health

## Abstract

Background: In the UK, breast cancer affects 1 in 8 women, accounting for 31% of cancers and 15% of cancer-related deaths in women. In 2023, London’s breast screening coverage was 56%, falling below the NHS target of 70%. This survey assesses psychological and behavioural factors impacting screening attendance. Methods: Using the Theoretical Domains Framework, an online survey including 15 behavioural factors was distributed through a market research company to women eligible for breast screening (aged 47–73) across London and Southeast and West England. Logistic regression was used to predict the impact of behavioural variables on history of attendance and intention to attend future screening opportunities. Results: Of the 922 respondents who returned the full survey, 88.6% intended to attend future screenings, and 88.1% reported previously attending screenings regularly. ‘Behavioural regulation’ had the strongest influence on past attendance (OR = 1.92, *p* < 0.001) and future intent (OR = 1.56, *p* = 0.003). Not intending to attend was linked to emotional consequences (OR = 0.68, *p* = 0.003) and environmental barriers (OR = 0.66, *p* < 0.001), where OR—Odds Ratio. Conclusions: This survey identifies behavioural factors influencing breast screening participation and screening intention, providing insights that may help design interventions to increase attendance rates.

## 1. Introduction

Cancer screening forms a central part of this public health policy (Department of Health and Social Care, 2018). Breast cancer is the most common cancer in the UK, with 1 in 8 women affected during their lifetime (Cancer Research UK, 2015). The 5-year survival rates for diagnosis at stage 1 and 2 are 98.2% and 89.5%, respectively, but this drops to 72.2% at stage 3 and 26.6% at stage 4 (Cancer Research UK, n.d.). Screening detects breast cancer at earlier stages when treatment is likely to be less invasive and more successful. Around 18,800 (34%) of breast cancer cases were diagnosed at stage 1 in England in 2018. By enabling earlier detection, it is estimated that the NHS breast screening programme saves 1300 lives per year (NHS, 2021).

Despite the benefits of breast cancer screening (BCS), attendance is far below the NHS target of 70% (NHS England, 2024). London uptake rates were 56% in 2023 and have been below recommended uptake levels since 2019/2020, with only very limited increases in uptake since there are socio-demographic inequalities in uptake (Douglas et al., 2016)—in London, the odds of attendance in the Indices of Multiple Deprivation (IMD) quintiles 1/2 is 11% lower than quintiles 4/5 (Jack et al., 2016), and the adjusted odds of attendance are almost 3 times higher amongst white British women than Asian women and 6 times higher than Black women (Jack et al., 2014). Those in the most deprived areas have 5-year survival rate that is 7.8% lower than those in the least deprived areas (Bhola et al., 2015).

There has been considerable work that has investigated the barriers and enablers of breast cancer screening participation and tested interventions to improve engagement (Haynes et al., 2023; Hofvind et al., 2023; Jarrar et al., 2023; Kue et al., 2024; Montero-Moraga et al., 2021; Nanda et al., 2022; Roh et al., 2023; Shoushtari-Moghaddam et al., 2023; Sniehotta et al., 2005; Wyatt et al., 2023). Previous work has highlighted logistical, cognitive, and emotional barriers (Bolarinwa & Holt, 2023; L. A. Marlow et al., 2015) that can impede attendance of mammographic screenings and several trials have used behavioural interventions to try to increase participation rates (Acharya et al., 2021), demonstrating varying degrees of effectiveness. Often these studies did not explain how their interventions were informed (Allgood et al., 2016; Offman et al., 2014). This study used a survey based on the Theoretical Domain’s Framework (TDF) (Cane et al., 2012) to assess the strength of psychological influences on the two outcome variables of history of attendance and intention to attend screenings in the future. Both variables are known predictors of future screening attendance (Allen et al., 2008; Lechner et al., 1997; Lo et al., 2015; Rutter, 2000; Velentzis et al., 2024; Wilson et al., 2021).

Michie et al.’s validated TDF is a comprehensive behavioural framework consolidating domains from 33 psychological theories (Cane et al., 2012). The updated version includes 14 domains, which can be used to inform the design of quantitative research such as surveys and questionnaires. Using the TDF also has benefits as it can be used with other behavioural tools such as the Behaviour Change Techniques Taxonomy (Michie et al., 2013) and the Theory and Techniques Tool (Johnston et al., 2020) to support the design of interventions which can appropriately address the identified barriers and facilitators. The TDF was applied to design a survey to measure behavioural barriers and enablers that may influence breast cancer screening participation and to identify the behavioural domains found to have a strong influence on attendance at screening, which should be addressed in future intervention.

## 2. Materials and Methods

### 2.1. Participants

Women aged 47–73 (breast screening age) in the London and Southwest and Southeast England area were invited to participate in the online survey through a third-party market research company (Bilendi, www.bilendi.co.uk) which hosts an online survey platform. Bilendi invited eligible women who are part of their panel to participate via email, as well conducting further recruitment by posting online adverts via social media to invite them to take part in the survey in exchange for a shopping voucher. Women outside of the age range, women who were not eligible for breast screening (e.g., women who had undergone a bilateral mastectomy), and men were excluded from the study. Incomplete surveys were treated as withdrawals and not included in the analysis. Seventy-eight women completed the pilot survey, then, following question removal (see “barriers and facilitators” section), a further 922 women returned the full survey, leading to a total sample size of 1000. The data were collected in 2016, and participants were reimbursed for their time through online tokens.

### 2.2. Measures

The survey (Supplementary Table S2) included outcome variables (past attendance and intention to screen), demographic and socio-economic characteristics, and constructs on barriers and facilitators to screening, developed using the TDF (Cane et al., 2012).

Past attendance was measured using three responses ‘Yes, always’, ‘Yes, sometimes’, and ‘No, never’. ‘Yes, sometimes’ and ‘No, never’ were grouped to create a binary variable containing an ‘infrequent or non-attenders’ group, compared to the ‘regular attenders’ group. Intention to attend future breast cancer screening when offered was measured using three responses ‘Yes’, ‘No’, and ‘I don’t know’. Both intention and past behaviour were included as outcomes to explore reasons for previous attendance and also any other reasons associated with whether they think they will attend in the future. Including intention meant we could also assess influences in women who had not previously been invited.

The survey included the following demographic characteristics: age group, region, level of education, employment, ethnicity, household income, and marital status.

The variables measuring barriers and facilitators to breast screening were based on the Theoretical Domains Framework (TDF) and informed by a review of the literature on determinants of screening (Bosgraaf et al., 2014). Variables contained between two and four items, with responses on a 7-point scale. Some items were reversed scored. Item wording was developed by the authors based on the TDF guidance and review of the literature. A pilot survey was used to reduce the number of questions using Cronbach’s alpha (CA) analysis to test internal consistency. Of the 14 variables, 8 reached a CA of over 0.7 (acceptable) after the removal of 16 items in total. The *Social influences* variable was split in two (“descriptive norms”, which refers to the perceived screening behaviour of family and friends, and “injunctive norm”, which refers to what family and friends and GPs think about screening) and retested—subsequently both variables had a CA score over 0.7. The *Skills* variable only had a CA of 0.188, so it was removed. For the four domains that did not reach a CA of 0.7 from the pilot (*Beliefs about capability, Reinforcement, Memory, Behavioural regulation*), one new item was added to each domain to try to improve the CA scores in the full survey.

The final survey contained 15 behavioural variables, across 11 of the 14 domains from the TDF. The domain of *Intention* was included as a dependent variable. The domains not included in the survey were: *Skills, Social/professional role and identity*, and *Reinforcement*. Some of the TDF domains were subdivided into multiple different behavioural variables to address different aspects of determinants of breast screening, and the variables were given labels to reflect the determinant being measured.

The full list of behavioural variables grouped by TDF domain can be found in Table 1. A table with items used in each variable can be found in Supplementary Table S1.

## 3. Ethics and Consent

After reading the information page at the beginning of the survey, respondents were asked to tick a consent box to be included in the research. Informed consent was obtained from all subjects involved in the study.

Ethical approval was received from Imperial College Research Ethics Committee (IREC reference: 15IC2710). All study methods adhered to relevant guidelines and regulations.

## 4. Analysis

The mean response was calculated of all items in each variable. Box–Tidwell and multicollinearity tests were performed for all continuous variables to be included in the regression to ensure test assumptions were met.

The association between the psychological variables and the two dependent variables ‘history of attendance’ and ‘intention to attend’ were tested using two separate backwards stepwise logistic regressions. An indication of how each domain predicts past attendance is indicated by the odds ratios (OR), which show the change in likelihood of previously having attended screening associated with a 1 standard deviation (SD) change in the domain score. The analyses were then repeated and adjusted for demographic and socio-economic variables.

Analysis was conducted using IBM SPSS Statistics 22.

## 5. Results

### 5.1. Sample

The sample of 1000 women had a mean age group of 55–59. Over 90% of the sample described themselves as white British, 3% as ‘white Other’, and 5.1% from another ethnic minority group. Demographics for the sample can be found in Table 2.

### 5.2. Survey Validity

Survey domains for the final survey were tested using Cronbach’s alpha (CA) for internal consistency. Eleven domains showed a high level of internal consistency with CA coefficient of >0.7 (see Table 1 above).

Four domains did not meet the 0.7 cutoff for CA: *Beliefs about capability* (0.63), *Beliefs of test reliability* (0.63), *Goals-future health* (0.6), and *Impact of dread* (0.53). As it was not possible to remove items to improve these scores, the domains were still included in the analysis.

### 5.3. Assumption Testing

The assumption of linearity was met for all variables according to the Box–Tidwell procedure (Box & Tidwell, 1962). From tests of multi-collinearity, several domains had potentially problematic multicollinearity with other domains. While multicollinearity can inflate standard errors and potentially render beta coefficients statistically insignificant in regression analyses, it does not necessarily invalidate the results. Therefore, it was decided to proceed to the regression analysis accepting the existing multicollinearity as any statistical significance of domains identified would be conservative estimates and, therefore, reliable.

### 5.4. Influences of Past Attendance

Of those previously invited to breast screening (*n* = 820), 88.1% (*n* = 722) reported having previously attended regularly.

No significant relationship was found between demographic and socio-economic factors and self-reported history of attendance.

The results of the backwards-stepwise logistic regression are shown in Table 3. The domains *Behavioural regulation, Screening priority, Value, Social norms—descriptive: family and friends*, and *Memory* had ORs over 1, indicating that women who score highly in these domains are more likely to have regularly attended breast screening in the past. *Behavioural regulation* (OR = 1.92, 95% CI 1.45–2.53, *p* < 0.001) and *Screening priority* (OR = 1.56, 95% CI 1.16–2.10, *p* = 0.003) were the two strongest enablers. *Environmental Context and resources* barriers (OR = 0.66, 95% CI 0.53–0.82, *p* < 0.001), *Beliefs about capability* (OR = 0.67, 95% CI 047–0.96, *p* = 0.029), or perceiving negative *Emotional impact of screening* (OR = 0.68, 95% CI 0.53–0.88, *p* = 0.003) had an influence on non-attendance.

### 5.5. Influencers of Intention to Attend in the Future

A total of 88.6% (*n* = 817) of survey respondents answered ‘Yes’ when asked about their intention to attend future breast cancer screening when offered, 11.4% (*n* = 105) answered ‘No’, and 0% ‘I don’t know’. Of the demographic and socio-economic factors, marital status was the only factor significantly associated with intention to attend breast screening. Women who were widowed were almost eight times more likely to attend breast screening compared to single women (OR = 7.8, 95% CI 1.16–52.62, *p* = 0.035). However, only 5.9% of the survey population were widowed.

The results of the logistic regression are shown in Table 4. *Behavioural regulation*, *Screening priority, Value, Social norms—injunctive—GP*, and *Goals-future health* had ORs above 1, indicating they are associated with intention to attend screening. The two strongest influences on intention to attend were *Behavioural regulation* (OR = 2.48, 95% CI 1.81–3.40, *p* < 0.001) and *Value* (OR = 2.07, 95% CI 1.54–2.79, *p* < 0.001). *Beliefs about Capability* (OR = 0.66, 95% CI 46–0.95, *p* = 0.024) and *Emotional impact of screening* (OR = 0.57, 95% CI 0.43–0.77, *p* < 0.001) had ORs below 1, indicating that they predicted intention to not attend screening.

When including demographic and socioeconomic factors, marital status significantly affected the intention to attend breast screening. Although the ORs did not shift considerably within the psychological domains, women who were widowed are almost eight times more likely to attend breast screening compared to single women (OR = 7.8, 95% CI 1.16–52.62, *p* = 0.035).

## 6. Discussion

This study aimed to develop a comprehensive survey tool to measure determinants of breast cancer screening uptake using the TDF. The strongest facilitators of previous attendance were *Behavioural regulation* and *Screening priority*. The strongest barriers to previous regular screening attendance were *Environmental factors*, *Beliefs about capability*, and the *Emotional impact of attending screening*.

*Behavioural regulation* was identified as the strongest facilitator of both outcome variables (previous regular attendance and intention to attend future screening opportunities) with similar impacts on both. This supports the evidence that people who make plans to attend health appointments are more likely to attend (Clemow et al., 2000; Michie et al., 2004; Rutter et al., 2006).

The second strongest domain predicting previous attendance at breast cancer screening was *Screening priority*. This domain consisted of items measuring the importance placed on making time for breast cancer screening relative to other life demands and is aligned with the TDF domain of *Goals*. Previous work has indicated that one’s ability to prioritise a behaviour can be heavily influenced by stress and social support. Further, evidence has found a positive relationship between stress and socio-economic status (SES). Therefore, the ability to prioritise preventative behaviours including cancer screening may be affected by underlying levels of stress and SES—influencing behavioural outcomes. Therefore, *Screening priority* is a key domain not only in terms of predictive strength but also because of environmental factors which may affect the ability to prioritise screening. It is therefore possible that an intervention targeting screening priority may differentially affect more disadvantaged cohorts. Previous research has shown that women often report being ‘too busy’ as a barrier to screening attendance (Bolarinwa & Holt, 2023; L. Marlow et al., 2015; Public Health England, 2016; Verberckmoes et al., 2024)—these women are likely to have low *Screening priority* scores, making interventions targeting this domain promising. However, because these women are likely to experience higher levels of stress or are of lower SES, it is possible that a *Screening priority* intervention may not overcome underlying factors which determine the ability to prioritise a preventative behaviour.

It is already recognised that forgetting about screening appointments can affect uptake (Bosgraaf et al., 2014; Brown et al., 2013; Crump et al., 2000; Ekechi et al., 2014; Feldstein et al., 2011; Glanz et al., 1992). Trials have shown the effectiveness of interventions including simple reminders in the forms of letters, telephone calls, and SMS message reminders for breast, cervical, and bowel cancer screenings (Acharya et al., 2021; Allgood et al., 2016; Arcas et al., 2014; Duffy et al., 2017; Kerrison et al., 2015; Richards, 2019; Taplin et al., 2000). This is consistent with our finding that *Memory* was a key enabler to previous attendance.

The regression findings suggest that the *Descriptive social norms* (describing how other people behave) domain also predicted previous regular attendance, but did not seem to predict intention. This inconsistency is in line with previous work on social norms and screening. Evidence from bowel cancer screening shows that social norms may play a key role in both intention to attend and screening participation (Sieverding et al., 2010); however, the study also highlighted the importance of the information within the social norm affecting the direction of effect. The study presented both a ‘high’ and ‘low’ true social norm to study participants and found that both resulted in lower intention to attend and participation rates than in those who received no social norm messages. This was likely due to even the ‘high’ norm not being sufficiently high (65%) enough to change behaviour (Cialdini, 2003; Sieverding et al., 2010).

The strongest barriers to previous regular screening attendance were *environmental* factors, e.g., distance to screening, *Beliefs about* capability, e.g., I have control over whether I attend breast screening when invited, and the emotional impact of attending screening, e.g., screening is reassuring. It is already recognised that environmental factors such as transport (Bailly et al., 2023), ability to take time off work (Cavers et al., 2022), and distance to screening sites (Jensen et al., 2014; Maheswaran et al., 2006) can affect attendance. It is therefore expected that women with higher environmental barrier scores are less likely to attend screening. However, *Environmental barriers* was not a domain retained in the final regression for intention to screen as it was removed by the stepwise regression model. This is potentially because women who do not intend to attend screening are likely to have made a decision before being exposed to environmental barriers, which may further affect their decision. Women who do not intend to screen are likely to either object to screening, not feel that screening is valuable or to be affected by emotional factors. This is supported by the finding that the *Value* and *Emotional consequences* variables predicted both past attendance as well as intention to participate in the future. This is in alignment with existing evidence that shows that anxiety about the screening, fear of pain, and embarrassment are well-recognised barriers to screening attendance (Acharya et al., 2021; Aro et al., 2001; Bolarinwa & Holt, 2023; Consedine et al., 2004; Crump et al., 2000; Fayanju et al., 2014; Feldstein et al., 2011; Flynn et al., 2011; Jaafar Sidek et al., 2023; Lee & Schwartz, 2019; Trigoni et al., 2008; Whelehan et al., 2013). A high score in *Beliefs about capability* predicted both previous non-regular attendance in the past and not intending to attend in the future. This may be explained by optimism bias which suggests that humans have a tendency to overestimate the frequency of positive events as well as their own ability to change their behaviour. An example of this sort of bias is seen amongst smokers and vapers who frequently overestimate their own ability to stop smoking (Strombotne et al., 2021; Weinstein et al., 2004, 2005).

In the context of screening, it is possible that women feel they have a high level of control over attending screening even if they have not attended regularly in the past. Therefore, they score high in the controllability domain whilst also reporting not previously attending regularly. However, this explanation becomes problematic, when considering that the *Beliefs about capability* domain also strongly predicted non-intention to attend. This means that women who score highly in *Beliefs about capability* were less likely to intend to attend. In this case, it is possible that women who feel in complete control and do not intend either object or are making a conscious decision not to attend screening.

Evidence has previously linked socioeconomic status to rates of preventative health behaviours (Danner et al., 2008; Sheldon Cohen & Salonen, 1999; Svendsen et al., 2020; Wang et al., 2022) including cancer screening. Further work has explained these differences in the behaviour of people from different SES groups through a psychosocial model that suggests that low SES groups experience higher levels of life-stress and lower levels of social support (Wardle et al., 2000). However, research has also highlighted the importance of physical, cognitive, and emotional factors, such as the psychological domains examined in this survey, as mediators to preventative health behaviours (Acharya et al., 2021; Crump et al., 2000; Fawns-Ritchie et al., 2022; Feig et al., 2022; Flynn et al., 2011; Glanz et al., 1992; L. A. Marlow et al., 2015; Marvan et al., 2013; Prowse et al., 2024; Waller et al., 2009). Further evidence also suggests that such factors vary significantly by SES (Creavin et al., 2023; L. Marlow et al., 2015; Wardle et al., 2004; Wardle & Steptoe, 2003). Work by (Wardle et al., 2004) on colorectal screening found that cognitive barriers such as *Worry* and *Perceived risk and benefits* were able to explain levels of interest in screening to the extent that they mitigated the effect of SES within the model so that they become non-significant (Douglas et al., 2016). The results of the survey further support these results by Wardle et al. as the model for the dependent variable of ‘history of regular attendance’ was not altered by including variables that contribute to SES or demographics such as age, geographical region, level of education, employment characteristics, ethnicity, household income, and marital status. Further, the ORs for predicting intention to attend were only minimally altered through marital status, and no other SES variables remained in the model. The overall variance explained was also only minimally improved from 74.0% in the model with psychological domains alone compared to 75.7% in the model including psychological domains and demographics, indicating that psychological domains explain the majority of the variance in previous attendance and intention to attend. This does not mean that SES does not affect attendance or intention to attend but that the psychological domains in the model may account for different SES factors represented in the SES variables included in the analysis.

It is recognised in the literature that previous screening behaviour has a stronger influence on future behaviour than intention to carry out that behaviour (Danner et al., 2008; Ouellette, 1998), suggesting that domains found to predict previous regular breast screening attendance should be prioritised in interventions to improve breast screening attendance—especially *Behavioural regulation* and *Screening priority*. The intention–behaviour gap refers to the disconnect between an individual’s stated intentions and their actual behaviour (Sniehotta et al., 2005) and is an additional reason why past screening attendance may be a better predictor of future attendance than self-reported intention to attend.

## 7. Study Limitations and Strengths

This was a large survey based on a comprehensive framework of behavioural influences. The study quantitatively shows the influences on both past attendance and intention to attend breast cancer screening, and the findings can be used to inform the design of interventions to improve BCS uptake.

Despite trialling and adjusting the constructs included in each domain in a pilot and full survey, four of the final domains had suboptimal Cronbach’s Alpha coefficients: Impact of dread, Beliefs about capability, Beliefs of test reliability, and Goals-future health. Given the perceived importance of these domains, it was decided that they should nonetheless be included in the regression analysis. Several domains had potentially problematic multicollinearity with other domains. As multicollinearity can explain why significance was not reached, the statistical significance of domains from the regression analysis should be conservative estimates and, therefore, reliable. However, these limitations should be considered when interpreting the findings. Eighty-eight percent of respondents who had previously been invited for breast cancer screening reported having attended regularly, compared to the national coverage of 75.4% at the time recorded by the NHS breast screening programme. This suggests that it is possible survey respondents were overly optimistic in their memory of their previous attendance or subject to a social desirability bias to report higher rates of screening. On the other hand, it is possible that the sample was not representative, as women who have not attended screening in the past may be less likely to answer a survey about breast cancer screening leading to a selection bias in the sample. If past attendance and intention to attend was over reported, this would also lead to less variance in the outcome variables reducing the ability to detect determinants of attendance. As the data are now quite old, there may have been changes in the reasons for attendance, though there has not been another large survey of reasons for breast cancer screening in the UK since then.

There is also a possible selection bias in that we recruited respondents through an online market research company and the survey was in English only, meaning that women who responded were generally internet-literate and were likely to have a high proficiency of the English language. This may have been mitigated by the fact that over 55% of respondents lived in households with an annual gross income of less than £34,999 and over 66% had a technical vocational degree or lower as their highest level of, indicating that they are likely to represent a lower SES. Additionally, 90% of respondents were white British, which may have masked important differences between different groups as there was low variance in the sample, and women from ethnic minority groups are known to have lower uptake of Breast Cancer Screening. Some findings, such as the much higher likelihood of screening in women who are widowed, may be an artefact of the small numbers in that category.

Finally, as a purely quantitative study, it does not provide an in-depth understanding of the barriers and facilitators of screening. Qualitative work is recommended to explore influences on screening in more detail.

## 8. Conclusions

This study measures the influence of a set of theory-informed behavioural domains in predicting breast screening intention and previous screening behaviour, whilst adjusting for demographic and socioeconomic factors. It provides further evidence that psycho-social, modifiable factors psychological domains are likely to be stronger mediators in screening behaviour and intention than many demographic or socioeconomic factors. The strongest barriers to previous regular screening attendance were *environmental* factor, e.g., distance to screening. This indicates that communication interventions focusing on beliefs about capability and emotional impact of attending screening are likely to be most effective at improving uptake given that previous attendance is a better predictor of future attendance than intention to attend.

Further work should be undertaken to better understand how the influence of different barriers and enablers differ according to factors such as region, ethnicity, cultural beliefs, age, and level of deprivation to allow a more targeted approach to intervention design.

## Figures and Tables

**Table 1:** Final behavioural variables and their mean scores and Cronbach’s alpha. The items were assessed on a 7-point Likert scale. Where items were negatively phrased, their scales were reversed prior to analysis.

TDF Domain	Behavioural Variable	Variable Mean (SD)	Cronbach’s Alpha
Social influences	Social norms descriptive—family and friends	5.42 (1.22)	0.83
	Social norms injunctive—family and friends	5.98 (1.25)	0.72
	Social norms injunctive—GP	5.56 (1.34)	0.78
Emotion	Impact of dread	4.95 (1.41)	0.54
	Emotional impact of screening	5.06 (1.46)	0.79
Behavioural regulation	Behavioural regulation	5.91 (1.32)	0.84
Goals	Screening priority	5.58 (1.16)	0.87
	Goals-future health	5.80 (S1.21)	0.60
Beliefs about consequences	Beliefs of test reliability	4.81 (1.07)	0.63
	Value	6.29 (1.16)	0.94
Optimism	Perceived low risk of breast cancer	5.86 (1.18)	0.88
Environmental Context and resources	Environmental context and resources	5.16(1.66)	0.86
Beliefs about capability	Beliefs about capability	6.28 (0.92)	0.63
Knowledge	Knowledge	6.39 (0.78)	0.83
Memory, attention, and decision processes	Memory	6.11 (1.04)	0.71

Note. TDF = Theoretical Domain’s Framework.

**Table 2:** Sample demographics.

Variable	Value	*n*	%
Age group	47–49	120	13
	50–54	201	21.8
	55–59	191	20.7
	60–64	184	20
	65–69	166	18
	70–73	60	6.5
Region	London	183	19.8
	South East	437	47.4
	South West	302	32.8
Level of education	O-level/GCSE	281	30.5
	A-level/Secondary school Graduate	153	16.6
	Trade/Technical/Vocational Qualification	176	19.1
	Bachelor’s degree	180	19.5
	Master’s degree or postgraduate degree	71	7.7
	Doctorate degree	10	1.1
Employment characteristics	Professional or higher technical work	146	15.8
	Manager or Senior Administrator	159	17.2
	Junior Manager	80	8.7
	Non-managerial, non-manual work	129	14
	Foreman or Supervisor of Other Workers	30	3.3
	Skilled Manual Work	113	12.3
	Semi-Skilled or Unskilled Manual Work	96	10.4
	Other	163	17.7
	Have never worked	6	0.7
Ethnicity	White British	835	90.6
	White Other	28	3
	Black British	11	1.2
	Black Other	9	1
	Asian British	13	1.4
	Asian Other	4	0.4
	Mixed British	10	1.1
	Did not disclose	12	1.3
Household income before tax	Less than £24,999	343	37.2
	£25,000 to £34,999	172	18.7
	£35,000 to £49,999	134	14.5
	£50,000 to £74,999	88	9.5
	£75,000 to £99,999	25	2.7
	£100,000 to £149,999	10	1.1
	£150,000 or more	2	0.2
	Refuse/Don’t know	148	16.1
Marital Status	Single, never married	91	9.9
	Married, or domestic partnership	597	64.8
	Separated	19	2.1
	Divorced	161	17.5
	Widowed	54	5.9
Private mammograms	Yes	47	5.1
English as first language	Yes	885	96

**Table 3:** Backward stepwise logistic regression model—final step for ‘History of attendance’.

	OR	*p*-Value	95% CI for OR	
			Lower	Upper
**Domain**				
Beliefs about capability	0.67	0.029	0.47	0.96
Behavioural regulation	1.92	0.000	1.46	2.53
Screening priority	1.56	0.003	1.16	2.10
Value	1.51	0.002	1.16	1.97
Social norms—descriptive: family and friends	1.36	0.022	1.05	1.76
Environmental context	0.66	0.000	0.53	0.82
Perceived low risk of breast cancer	0.75	0.067	0.54	1.02
Emotional impact of screening	0.68	0.003	0.53	0.88
Memory	1.52	0.008	1.12	2.08
Constant	0.043	0.064		
Nagelkerke pseudo r^2^	62.70%			
Hosmer and Lemeshow test	χ^2^(8) = 9.65, *p* = 0.265			

Note. OR = Odds Ratio.

**Table 4:** Backward stepwise logistic regression models for ‘Intention to attend’ future screening rounds.

	Model 1				Model 2			
	OR	*p*-Value	95% CI		OR	*p*-Value	95% CI	
			Lower	Upper			Lower	Upper
**Domain**								
Beliefs about capability	0.66	0.024	0.46	0.95	0.61	0.009	0.42	0.88
Behavioural regulation	2.48	0.000	1.81	3.40	2.54	0.000	1.81	3.55
Screening priority	1.36	0.034	1.02	1.81	1.39	0.023	1.05	1.84
Value	2.07	0.000	1.54	2.79	2.12	0.000	1.56	2.87
Emotional impact of screening	0.57	0.000	0.43	0.77	0.56	0.000	0.41	0.75
Social norms—injunctive: GP	1.46	0.024	1.04	2.03	1.43	0.031	1.03	2.02
Goals-future health	1.61	0.019	1.08	2.40	1.77	0.007	1.17	2.67
**Marital Status**						0.033		
Single	-							
Married, Civil partnership	-				1.32	0.617	0.45	3.88
Separated					0.7	0.998	0.00	-
Divorced	-				0.51	0.251	0.16	1.62
Widowed	-				7.80	0.035	1.16	52.62
Constant	0.00	0.000			0.00	0.000		
Nagelkerke pseudo r^2^	74.00%				75.70%			
Hosmer and Lemeshow test	χ^2^(8) = 5.205, *p* = 0.735				χ^2^(8) = 6.976, *p* = 0.539			

## Data Availability

The raw data supporting the conclusions of this article will be made available by the authors on request.

## Supplementary Materials

The following supporting information can be downloaded at: https://www.mdpi.com/article/10.3390/bs15020209/s1, Table S1: Table of TDF domains mapped onto variable labels and survey items; Table S2: Pilot and full Survey questions.
