# Supplementary material for: A Population Survey on Barriers and Facilitators to Breast Cancer Screening Based on the Theoretical Domains Framework
Source: Behav Sci (Basel). 2025 Feb 14;15(2):209. doi: 10.3390/bs15020209 (PMC11851805; doi:10.3390/bs15020209)
Supplement: Supplementary file 1 [file behavsci-15-00209-s001.zip › behavsci-3391399-supplementary.pdf]

Table S1 - *Table of TDF domains mapped onto variable labels and survey items*

TDF Domain	Variable label	Items
Social influences	Social norms- descriptive: Family and friends	- In my group of friends most women about the age of 50 have regular mammograms<br>- In my family most women above the age of 50 have regular mammograms
	Social norms- injunctive: Family and friends	- Most people who are important to me think that I should attend breast screening when invited.<br>- The people in my life whose opinions I value would approve of my attending breast screening when invited
	Social norms- injunctive: GP	- My GP expects me to attend my screening mammogram appointment when invited<br>- Generally speaking I intend to do what my GP expects of me
Emotion	Impact of dread	- If I dread doing something I do it as soon as possible to get it out of the way<br>- I tend to put things off that I am dreading doing
	Emotional impact of screening	- Upsetting- reassuring<br>- I dread being invited for a screening mammogram<br>- I am worried about pain and comfort during a mammogram<br>- I expect to be very embarrassed during a screening mammogram
Behavioural regulation	Behavioural regulation	- I have a clear plan of how I will make time to attend my screening mammogram<br>- if/when invited, I have a clear plan of how I will attend my mammogram appointment
Goals	Screening priority	- Generally, there is something more urgent that needs my attention than making time for a screening mammogram<br>- Generally, there is something of a higher priority than making time for a screening mammogram
	Goals- future health	- I would make an effort to attend breast screening if the result could inform me of my future risk of developing breast cancer<br>- Attending screening mammogram is consistent with the importance I put on my personal health
Beliefs about consequences	Beliefs of test reliability	- A breast cancer might be missed (false negative)<br>- A breast cancer might be diagnosed when, really there is no cancer (false positive)<br>- A cancer may be diagnosed that might not need any treatment, or would not affect me in my lifetime
	Value	- Valuable – worthless<br>- Beneficial – harmful<br>- A priority – not important
Optimism	Perceived low risk of breast cancer	- If I have no breast symptoms, then attending breast cancer screening is unlikely to be beneficial<br>- If I examine my breast regularly then there is not much benefit to me from attending breast screening

		- A women of my age is unlikely to develop breast cancer
Environmental Context and resources	Environmental context and resources	- The distance of the screening centre from my home will affect whether I attend.<br>- Availability of transport (public or personal) to the screening centre might affect me attending<br>- If I am given an inconvenient appointment time, I might not attend
Beliefs about capability	Beliefs about capability	- I have complete control over whether I attend breast screening when invited<br>- It is completely up to me whether or not I attend breast screening when invited
Knowledge	Knowledge	- Breast screening can pick up cancer at an earlier stage<br>- If a breast cancer is treated early, survival is more likely<br>- Receiving a normal screening result will provide reassurance<br>- Not attending screening may lead to a breast cancer growing undetected for longer
Memory, attention and decision processes	Memory	- I have never accidentally missed a mammogram appointment because I forgot<br>- I never forget about routing health appointments<br>- I have previously missed my mammogram because I forgot about the appointment<br>- I always make a note of the time and date of any routine health appointments

Table S2- Pilot and full Survey questions

		Pilot Survey	Full Survey
**Elligibility questions**			
S1	Are you a woman?	✓	✓
S2	Which age group are you in?	✓	✓
S3	S3: Where do you live?	✓	✓
S4	Which borough are you based	✓	✓
**Survey Questions**			
1	I have been invited for a screening mammogram before.	✓	✓
2	If/When I am invited for a screening mammogram I intend to attend.	✓	✓
2.2	If/When I am invited for a screening mammogram I will definitely attend?	x	✓
3	I attend private mammograms	✓	✓
4	In the past I have attended a screening mammogram whenever I have been invited.	✓	✓
5	How many times have you attended a screening mammogram?	✓	✓
6	In the past, I have attended/taken part in other types of cancer screening whenever I am invited (e.g. cervical screening/bowel cancer screening)	✓	✓
7	Do you believe that there are any advantages of attending breast screening when invited?	✓	x
8	Do you believe that there are any disadvantages of attending breast screening when invited?	✓	x
9	Is there anything else you associate with you attending breast screening?	✓	x
10	To me personally, breast cancer screening is: Valuable - Worthless	✓	✓
11	To me personally, breast cancer screening is: Unpleasant - Pleasant	✓	x
12	To me personally, breast cancer screening is: Beneficial - Useless	✓	✓
13	To me personally, breast cancer screening is: Tolerable - Intolerable	✓	x
14	To me personally, breast cancer screening is: Bad - good	✓	x
15	To me personally, breast cancer screening is: Convenient - inconvenient	✓	x
16	To me personally, breast cancer screening is: Irrelevant - relevant	✓	x
17	To me personally, breast cancer screening is: Upsetting - Reassuring	✓	✓
18	To me personally, breast cancer screening is: Top priorty - unimportant	✓	✓
19	Breast screening can pick up cancer at an earlier stage. - Below are some statements relating to women's beliefs about benefits and disadvantages of breast screening. Please read each statement below and answer according to how much you agre	✓	✓
20	If a breast cancer is treated early, survival is more likely. - Below are some statements relating to women's beliefs about benefits and disadvantages of breast screening. Please read each statement below and answer according to how much you a	✓	✓
21	Receiving a normal screening result will provide reassurance. - Below are some statements relating to women's beliefs about benefits and disadvantages of breast screening. Please read each statement below and answer according to how much you a	✓	✓
22	A breast cancer might be missed by screening. - Below are some statements relating to women's beliefs about benefits and disadvantages of breast screening. Please read each statement below and answer according to how much you agree or disagree	✓	✓
23	A breast cancer might be diagnosed from the screening, when really there is no cancer. - Below are some statements relating to women's beliefs about benefits and disadvantages of breast screening. Please read each statement below and answer ac	✓	✓
24	Not attending screening may lead to a breast cancer growing undetected for longer. - Below are some statements relating to women's beliefs about benefits and disadvantages of breast screening. Please read each statement below and answer accord	✓	✓
25	Mammograms involve a dose of radiation that might in itself cause harm or a cancer. - Below are some statements relating to women's beliefs about benefits and disadvantages of breast screening. Please read each statement below and answer acc	✓	x
26	A cancer may be diagnosed by screening that might not have needed any treatment, or would not affect me in my lifetime. - Below are some statements relating to women's beliefs about benefits and disadvantages of breast screening. Please read e	✓	✓
27	An abnormal screening result could make me anxious about a cancer diagnosis. - Below are some statements relating to women's beliefs about benefits and disadvantages of breast screening. Please read each statement below and answer according to	✓	✓
28	I do not want to attend breast screening as I may suffer unnecessary anxiety from an abnormal test result. - Please give your honest response to the statements below about breast screening:	✓	x
29	I do not want to attend breast screening as I may end up having unnecessary treatment / tests, for an abnormal mammogram result that may not turn out to be cancer. - Please give your honest response to the statements below about breast screening	✓	✓
30	My previous breast screening mammograms have been negative. I am therefore unlikely to develop breast cancer - Please give your honest response to the statements below about breast screening:	✓	✓
31	I am physically able to attend screening. - Please give your honest response to the statements below about breast screening:	✓	x
32	I dread being invited for a screening mammogram. - Please give your honest response to the statements below about breast screening:	✓	✓
33	I have complete control over whether I attend breast screening when invited. - Please give your honest response to the statements below about breast screening:	✓	✓

34	Most people who are important to me think that I should attend breast screening when invited. - Please give your honest response to the statements below about breast screening:	✓	✓
35	The people in my life whose opinions I value would approve of my attending breast screening when invited. - Please give your honest response to the statements below about breast screening:	✓	✓
36	My GP expects me to attend my screening mammogram appointment when invited. - Please give your honest response to the statements below about breast screening:	✓	✓
37	Generally speaking I intend to do what my GP expects of me. - Please give your honest response to the statements below about breast screening:	✓	✓
38	If I dread doing something I do it as soon as possible to get it out of the way. - Please give your honest response to the statements below about breast screening:	✓	✓
39	It is completely up to me whether or not I attend breast screening when invited. - Please give your honest response to the statements below about breast screening:	✓	✓
40	I think it would be difficult to attend breast screening due to high demands on my time from family or work. - Please give your honest response to the statements below about breast screening:	✓	x
41	The distance of the screening centre from my home will affect whether I attend. - Please give your honest response to the statements below about breast screening:	✓	✓
42	I plan to attend a mammogram in the future but not right now. - Please give your honest response to the statements below about breast screening:	✓	✓
43	I tend to put things off that I am dreading doing. - Please give your honest response to the statements below about breast screening:	✓	✓
44	Availability of transport (public or personal) to the screening centre might affect me attending. - Please give your honest response to the statements below about breast screening:	✓	✓
45	If I am given an inconvenient appointment time, I might not attend. - Please give your honest response to the statements below about breast screening:	✓	✓
46	I have never accidently missed a mammogram appointment because I forgot - Please give your honest response to the statements below about breast screening:	x	✓
46	If invited today, choose when you would prefer to attend screening:	✓	x
47	Are there people or types of people who would approve of you attending a screening mammogram?	✓	x
48	Are there people or types of people who would disapprove of you attending a screening mammogram?	✓	x
49	In my opinion breast cancer is generally very common. - Please indicate how much you agree with the statements below about the risk of breast cancer,	✓	✓
50	The likelihood of a woman like me developing breast cancer in my lifetime is low. - Please indicate how much you agree with the statements below about the risk of breast cancer,	✓	✓
51	If I have no breast symptoms, then attending breast cancer screening is unlikely to be beneficial. - Please indicate how much you agree with the statements below about the risk of breast cancer,	✓	✓
52	If I examine my breasts regularly then there is not much benefit to me from attending breast screening. - Please indicate how much you agree with the statements below about the risk of breast cancer,	✓	✓
53	A woman of my age is unlikely to develop breast cancer. - Please indicate how much you agree with the statements below about the risk of breast cancer,	✓	✓
54	A woman with my family's history of breast cancer is unlikely to develop breast cancer. - Please indicate how much you agree with the statements below about the risk of breast cancer,	✓	x
55	I would like to better understand my personal risk of getting breast cancer. - Please indicate how much you agree with the statements below about the risk of breast cancer,	✓	✓
56	I would make an effort to attend breast screening if the result could inform me of my future risk of developing breast cancer. - Please indicate how much you agree with the statements below about the risk of breast cancer,	✓	✓
57	I am worried about pain and discomfort during a mammogram. - Please indicate how much you agree with the statements below about the risk of breast cancer,	✓	✓
58	I am so worried about pain and discomfort that it might prevent me from attending. - Please indicate how much you agree with the statements below about the risk of breast cancer,	✓	x
59	I expect to be very embarrassed during a screening mammogram. - Please indicate how much you agree with the statements below about the risk of breast cancer,	✓	✓
60	I am so worried about being embarrassed, that it might prevent me from attending. - Please indicate how much you agree with the statements below about the risk of breast cancer,	✓	x
61	Attending screening mammograms is consistent with the importance I put on my personal health. - Please indicate how much you agree with the statements below about the risk of breast cancer,	✓	✓
62	For me personally, attending breast screening is very easy. - Please indicate how much you agree with the statements below about the risk of breast cancer,	x	✓
62	An average woman's chances of developing breast cancer in her lifetime is:	✓	
63	Do you know of any reasons why you might be less likely than the average woman to develop breast cancer?	✓	x
64	Do you know of any reasons why you might be more likely than the average woman to develop breast cancer?	✓	x
65	Has a blood relative close to you e.g. mother or sister ever had an abnormal screening mammogram?	✓	✓
66	Have you ever had an abnormal screening mammogram?	✓	✓
67	Has a blood relative close to you e.g. mother or sister ever been diagnosed with breast cancer?	✓	✓
68	Has a close friend ever been diagnosed with breast cancer?	✓	✓
70	Have you ever been diagnosed with breast cancer?	✓	✓
71	Have you ever been diagnosed with any other type of cancer?	✓	✓
73	Generally, there is something more urgent that needs my attention than making time for a screening mammogram. - Please indicate how much you agree with the statements below about the risk of breast cancer	✓	✓
74	Generally, there is something in my life of higher priority than making time for a screening mammogram. - Please indicate how much you agree with the statements below about the risk of breast cancer	✓	✓
75	I never forget about routine health appointments. - Please indicate how much you agree with the statements below about the risk of breast cancer	✓	✓

76	I have previously missed my mammogram because I forgot about the appointment. - Please indicate how much you agree with the statements below about the risk of breast cancer	✓	✓
77	In my group of friends most women above the age of 50 have regular mammograms. - Please indicate how much you agree with the statements below about the risk of breast cancer	✓	✓
78	In my family most women above the age of 50 have regular mammograms. - Please indicate how much you agree with the statements below about the risk of breast cancer	✓	✓
79	I keep track of my mammogram results. - Please indicate how much you agree with the statements below about the risk of breast cancer	✓	x
80	I have a clear plan of how I will make time to attend my screening mammogram. - Please indicate how much you agree with the statements below about the risk of breast cancer	✓	✓
81	Attending my mammogram is something I do without thinking. - Please indicate how much you agree with the statements below about the risk of breast cancer	✓	x
82	Receiving a text message to remind me of my breast screening appointment would be useful. - Please indicate how much you agree with the statements below about the risk of breast cancer	✓	✓
82	If/when invited, I have a clear plan of how I will attend my mammogram appointment. - Please indicate how much you agree with the statements below about attending breast screening and routine health appointments	x	✓
82	I always make a note of the time and date of any routine health appointments. - Please indicate how much you agree with the statements below about attending breast screening and routine health appointments	x	✓
85	What is the highest level of school/education you have obtained?	✓	✓
86	What is your marital status	✓	✓
87	Do any children aged 12 or under live in your household?	✓	✓
88	Do any adolescents (aged 13-17 years old) live in your household?	✓	✓
89	Are any members of your household aged 65 or over?	✓	✓
90	Are any members of your household disabled or have a physical handicap?	✓	✓
91	Is English your first language?	✓	✓
92	How would you describe your ethnicity	✓	✓
93	How many hours per week do you do paid employment?	✓	✓
94	What was your total household income before taxes in the last 12 months?	✓	✓
95	How often have you changed your address in the past 10 years?	✓	✓
96	How often have you changed your GP in the past 10 years?	✓	✓
97	How often have you changed your mobile phone number in the past 10 years?	✓	✓
98	Have you been invited for more frequent screening based on your family history or personal risk (normal screening is offered every 3 years)	✓	✓
